# Characterization and Association of the Missing Ventral Tubercle(s) from the Sixth Cervical Vertebra and Transpositions on the Ventral Surface of the Seventh Cervical Vertebra in Modern *Equus ferus caballus*

**DOI:** 10.3390/ani14121830

**Published:** 2024-06-20

**Authors:** Sharon May-Davis, Pamela Blades Eckelbarger, Diane Dzingle, Elle Saber

**Affiliations:** 1 Canine and Equine Research Group, University of New England, Armidale, NSW 2351, Australia; 2 Equus Soma—Equine Osteology and Anatomy Learning Center, Aiken, SC 29805, USA; eqsoma71@gmail.com (P.B.E.); dldzingle@gmail.com (D.D.); 3 Biological Data Science Institute, Australian National University, Canberra, ACT 2601, Australia; elle.saber@anu.edu.au

**Keywords:** transverse foramen, equine complex vertebral malformation (ECVM), caudal ventral tubercle (CVT), cranial ventral tubercle (CrVT), seventh cervical vertebra (C7), sixth cervical vertebra (C6), transposition

## Abstract

**Simple Summary:**

Congenital malformations of the equine caudal cervical vertebrae (C6 and C7) were first reported in the 1800s. However, by the 21st century these findings were no longer incidental, especially with studies reporting breed percentages as high as 38%. Therefore, this study examined the association between C6 osseous specimens (*n* = 85) demonstrating the congenital malformation of either a partial unilateral or bilateral absent ventral process and the transposition on the ventral surface of sequential C7s (44/85). The aim was to determine if the absent morphology on C6 corresponded to the transposition on C7 and in some cases replication of a transverse foramen (33/44). Findings revealed the larger morphological absence on C6 generated a stronger dependency for transposition on C7 that included the replication of a transverse foramen. The transposition on C7 was characterized by either a complete or incomplete likeness of the C6 ventral process. Complete transpositions on C7 appeared as a normal C6 ventral process, while incomplete transpositions appeared in most as a cranial ventral tubercle (CrVT). When complete bilateral transpositions were demonstrated, the morphology of C7 resembled C6. These findings are relevant to the reading of radiographic images and potential clinical findings.

**Abstract:**

In recent years, equine complex vertebral malformation (ECVM) has been of concern in the equine community, with studies identifying numerous associative morphological variations. Here, we examine the morphological association between C6 and C7 for dependency in ECVM cases, where the partially absent ventral process of C6 transposes on the ventral surface of C7. A C6 ventral process presents two tubercles, one cranial (CrVT) and one caudal (CVT). In this study, the C6 osseous specimens (*n* = 85) demonstrated a partial or completely absent CVT (aCVT) graded 1–4 that often extended cranially creating a partially absent cranial ventral tubercle (aCrVT) graded 1–3. In the 85 C6 osseous specimens examined, the corresponding C7s demonstrated either a complete or incomplete transposition of the ventral process from C6 in 44/85, with 30/44 replicating a transverse foramen. A strong statistical dependency existed between C6 grade 4 aCVTs and grades 1–3 aCrVTs and C7 transpositions with replicated transverse foramen. Sidedness was also demonstrated, where a left sided absent C6 associated with transposition on the left ventral surface of C7. This likewise applied to right sidedness and most bilateral cases. These findings might benefit practitioners when radiographing the extent of the ECVM configuration in patients presenting caudal cervical pain.

## 1. Introduction

Since the earliest known ancestor of modern *Equus ferus caballus*, *Hyracotherium,* the cervical vertebrae in the family Equidae have retained a similar primordial morphology throughout its evolutionary history [1,2,3,4]. As with most mammals, the equine neck comprises seven cervical vertebrae that are defined by three distinct modules. The characterization of each module is governed by vertebral shape (typical or atypical) and function, notably cranial (C1 and C2—atypical), mid (C3 to C5—typical), and caudal (C6 and C7—atypical) [4,5,6,7]. Until recently, congenital malformations of the cervical vertebrae were considered rare [8,9], even though incidental findings of anomalous variations were described by early anatomists in the caudal module [10]. However, by 2014 quantitative studies began to reveal the extent of C6 and C7 congenital malformations held greater than expected percentages in multiple breeds of horse. For example, Thoroughbreds (≥38%), Warmbloods (≥30%), Quarter Horses (≥23%), and to a lesser extent other breeds, such as Arabians (≥10%) [11,12,13,14,15,16,17]. As the current studies primarily focused on modern *E. ferus caballus*, two separate studies examined the C6 and C7 vertebrae in three extant sister taxa, Dutch Koniks (descendants of *E. ferus ferus*) and museum collections housing specimens of pre-domestic equids. The findings revealed that none of the randomly selected extant equids nor museum specimens were affected by the anomalous variations in C6 and C7 described in modern *E. ferus caballus* [18,19].

First reports relating to the anomalous morphology of C6 and C7 began with descriptions by early anatomists, for example, “C6 with a prolongation deficient in the transverse process and C7 with an additional prolongation” and “asymmetric C6 with bicuspid transverse processes on one side with the normal tricuspid on the other” (Hussou and Lesbre, respectively, in Chauveau 1908) [10]. May-Davis (2014) described a similar morphology from gross observation, noting an absent caudal ventral tubercle of C6 (either unilateral or bilateral) could transpose onto the ventral surface of C7 (either unilateral or bilateral) [12]. Thereafter, Veraa et al. (2020) coined the term Caudal Cervical Vertebral Morphological Variation from computed tomography scans [20], followed by the radiographic observations described as Equine Caudal Cervical Morphologic Variations (ECCMV) by Gee (2020) [21]. All three studies described the same C6 and C7 osseous malformations. Associative studies in 2015 and 2017 revealed these anomalous variations often coincided with other congenital anomalies, such as musculature (*longus colli* and *scalenus ventralis*), nerves (brachial plexus and phrenic), and sternal ribs (first and second) [22,23]. Similar combinations of gross osseous morphologies were reported in a syndrome known as Complex Vertebral Malformation (CVM) in Holstein cattle [24]; hence, it was deemed appropriate to assign Equine Complex Vertebral Malformation (ECVM) to the equine anatomical equivalent [25]. The distinction between ECVM and ECCMV is the former encompasses both osseous and soft tissue abnormalities (thus “complex”) in the cervicothoracic region of which the caudal cervical vertebrae (C6 and C7—ECCMV) are an integral part.

Although the literature might present varied anatomical nomenclature concomitant with C6, the descriptive terminology is well established. With this in mind, the normal morphology of C6 has specialized bilateral ventral processes that are atypical in the axial skeleton [18,25,26]. These bony projections are separate and distal to the transverse processes and appear as tube-like structures that extend along the left and right ventral borders of C6 in a craniocaudal orientation [18,25]. Each ventral process is further differentiated, morphologically, into a cranial ventral tubercle (CrVT) and a caudal ventral tubercle (CVT) [18,25]. Both regions serve as anchor sites for the *longus colli* muscle [22,26,27]. In ECCMV and ECVM horses, one congenital malformation is constant; that is, C6 presents a partial or completely absent CVT [12,13,14,15,16,17,18,25]. Initially, studies examining the congenital malformations of C6 described a unilateral or bilateral morphological change to the CVT, wherein sections of one or both tubercles are absent (aCVT) [10,11,12,13,14,16,17,18,25]. More recently, May-Davis et al. (2023) described multiple morphological characterizations of the C6 malformation that included not only the CVTs but, in some horses, a continuation of the absent tubercle to include portions of the CrVT (aCrVT) [25].

Furthermore, horses with an aCVT, whether partial or complete, could transpose one or both tubercles onto the ventral surface of C7 and replicate the transverse foramen from C6 [14,15,16,17,18,19,28]. Some authors have described this transposition on C7 as a “protuberance” [29], while others referred to it as a “transposed CVT from C6” [12], “transposed ventral lamina from C6” [16], or a “malformation with anomalous shape of C7” [17]. To date, studies describing the corresponding replication of the transverse foramen onto C7 [12,13] have not identified an associative frequency with the aCVT of C6, the presence of a transposition on C7, nor similarity in size. In general, authors agree on the terminology of the foramen with a few modern variants—arterial foramen or transverse foramen—while the Latin *foramen transversarium* is more commonly used in veterinary and older texts [26,29,30]. The significance here is that in the normal state, paired transverse foramen are present from C1 to C6 but are not present in the normal morphology of C7 [26], thus raising concerns as to whether the associative blood vessel is also deviated as per the *longus colli* and *scalene ventralis* muscles previously described in ECVM [22,23].

To date, evidence suggests the anomalous C7 is dependent on the aCVT of C6 [11,12,13,14,15,16,17,18,20,21,22,23], yet the morphological features of the transposition(s) on the ventral surface of C7 have not been fully examined or described in *E. ferus caballus*, nor has the intermittent replication of one or both transverse foramen onto C7. Therefore, the primary objective of this observational study is to describe, characterize, and analyze the morphological variations in the transpositions on the ventral surface of C7 to determine if a special association exists between the grades of C6 aCVT, as described by May-Davis et al. (2023) [25], and transposition(s) on C7. Additionally, we will report our observations on the presence and frequency of replicated transverse foramen on C7.

## 2. Materials and Methods

### 2.1. Ethical Statement

No horses were euthanized for the purpose of this study and observational research was conducted postmortem on osseous specimens of C6 and C7 with owner permission from private collections, educational facilities, and one private research facility.

### 2.2. Terminology

Primary nomenclature was derived from Getty [26] and the previous literature describing the grading of the aCVT of C6 and transposition of the aCVT onto the ventral surface of C7 [14,15,16,17,18,19,25,29].

### 2.3. Materials

To be eligible for the study, it was a prerequisite that C6 must demonstrate the congenital malformation of either a partial unilateral or bilateral absent ventral process with corresponding C7 from crossbreeds and purebreds of *E. ferus caballus.* It was not essential for C7 to demonstrate a congenital malformation. In addition, C6 and C7 osseous specimens had to demonstrate minimal damage with clear structural definition for the grading of the C6 aCVT and subsequent examination of C7.

Ten facilities housing suitable osseous specimens granted access to their collections—seven private, one research, one college, and one university (outlined in acknowledgements).

For comparative research purposes, the ventral surface of one normal C7 from a 19-year-old Australian Stock Horse male was used for this study. Eighty-five C6 osseous specimens with aCVTs (122 aCVTs from a possible 170 normal CVTs) and their associative C7s were examined. The specimens were sourced from nine countries: Australia (*n* = 48); United States of America (*n* = 20); Japan (*n* = 5); the Netherlands (*n* = 3); United Kingdom (*n* = 4); New Zealand (*n* = 2); Belgium (*n* = 1); Ireland (*n* = 1); Sweden (*n* = 1). Nine breeds (not inclusive of crossbreds) were represented by Thoroughbreds (*n* = 42); Warmbloods (*n* = 15); Australian Stock Horses (*n* = 6); Standardbreds (*n* = 5); Appaloosas (*n* = 3); Quarter Horses (*n* = 3); Friesians (*n* = 1); Irish Sport Horses (*n* = 1); Riding Ponies (*n* = 3); and Crossbreds (*n* = 6). The age by year ranged from stillborn to 30. The study consisted of 54 males and 31 females. Individual details are reported in Supplementary Materials Table S1.

Photographs were acquired using a NEEWER^®^ White Soft Box (Neewer Technology Co. Ltd., Shenzehen, China) 32 × 32-inch (81.3 × 81.3 cm) photobooth with a Canon EOS RP camera (Canon Inc., Tokyo, Japan) and 50 mm focal length lens. The camera was fixed to a NEEWER^®^ Mini Travel Tabletop Tripod (Neewer Technology Co. Ltd., Shenzehen, China) with a height range of 24 inches (60.9 cm) above normal desk height. Four NEEWER^®^ T120 LED (Neewer Technology Co. Ltd., Shenzehen, China) lights set at 5400 kelvin were utilized for lighting of the specimens. Only ventral and left lateral photographs were acquired for the study.

### 2.4. Methods

From the ventral view of C6, the anomalous characterization of the aCVT and aCrVT were examined and graded according to the protocols previously established by May-Davis et al. (2023) [25]. The ventral surface of the corresponding C7 to each anomalous C6 was then examined for three morphological presentations: complete transposition of a ventral process consistent with that found on a normal C6; incomplete transposition of a ventral process (consistent with either a CVT or CrVT as per that found on a normal C6); or no transposition. To qualify for a complete transposition on C7, the morphology must be representational of a combined CrVT and CVT as per a normal C6, with the CVT aligning with or passing the caudal border of the vertebral body. An incomplete transposition represents a partial ventral process on C7 that can be either a CrVT and or CVT. In these osseous specimens, the defining characteristic between incomplete and complete transposition is the alignment of the CVT with the caudal border of C7.

**Figure 1:**
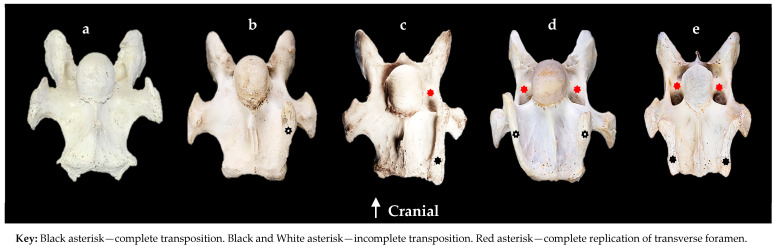
The ventral and cranio-ventral view of the seventh cervical vertebra. (**a**) Normal 19-year-old Australian Stock Horse male. (**b**) Tb 34—left side incomplete transposition with no replication of the transverse foramen. (**c**) App 2—left side complete transposition with a left side replication of the transverse foramen. (**d**) Tb 35—bilateral incomplete transpositions with bilateral replication of the transverse foramen. (**e**) App 1—bilateral complete transpositions with bilateral replication of the transverse foramen.

Furthermore, in all corresponding C7 osseous specimens, examination for the replication of a C6 transverse foramen was conducted. The presentations are recorded as either complete, normal in size compared to the transverse foramen in C6 or incomplete, which is noticeably smaller than normal or where it only partially extends through the vertebral body (Figure 1).

Observations of the anomalous C6 and C7 osseous specimens were conducted by the following authors—Sharon May-Davis (*n* = 65) and jointly Sharon May-Davis, Pamela Blades Eckelbarger, and Diane Dzingle (*n* = 20).

### 2.5. Statistical Analysis

The strength of association between each pair of variables was calculated using Cramer’s V (a common measure of the strength of association between two nominal variables). The statistical significance of this association was assessed using Fisher’s exact test. A Bonferroni correction was applied in evaluating statistical significance.

To study the associative structure between vertebral malformations jointly, multiple correspondence analysis (MCA) was used. MCA is an extension of correspondence analysis for more than two categorical variables simultaneously; it is used to estimate a low-dimensional approximation of the variables to allow for visualization.

All statistical analysis was conducted in R version 4.2.2 (R Core Team, Vienna, Austria). The tidyverse packages, including janitor [31], were used for data analysis processing. Pairwise associations were calculated with visual categorical data (vcd) package [32] and MCA with a FactoMineR package [33].

## 3. Results

Eighty-five anomalous C6 osseous specimens matching the criterion with sequential C7s were examined for associations between the two vertebrae. Characterizations of the morphology and analysis were further examined and reported here.

### 3.1. Morphological Variations in C6

In a normal C6 osseous specimen, two ventral processes should be evident. However, after examining the 85 anomalous C6 osseous specimens matching the criterion of this study, 122/170 ventral processes revealed differing morphology, with an aCVT evident in bilateral (*n* = 37), left unilateral (*n* = 34), and right lateral (*n* = 14) morphology. Of the 122 aCVTs, 60 included an aCrVT with individual results recorded in the Supplementary Materials Table S1.

### 3.2. Corresponding Morphological Variations of C7

Of the 85 anomalous C6 osseous specimens, 44 (52%) corresponding C7s presented 58 transpositions in bilateral (*n* = 14), left unilateral (*n* = 23), and right lateral (*n* = 7) morphology. These transpositions were similar to a complete or incomplete C6 ventral process, with 30/44 C7s replicating a transverse foramen in bilateral or unilateral morphology.

#### 3.2.1. Descriptive Morphology of a C7 Complete Transposition

A complete transposition resembles the ventral process of C6 on the ventral surface of C7. In these specimens, the transposition extended to the caudal border in close proximity to the *fovea costalis caudalis* (Figure 1c,e). It was evident in 14/44 C7s, representing 19/58 transpositions. The configurations were bilateral (*n* = 5), left unilateral (*n* = 8), and right unilateral (*n* = 1).

#### 3.2.2. Descriptive Morphology of a C7 Incomplete Transposition

An incomplete transposition of the ventral process resembles either the CrVT or CVT of C6 on the ventral surface of C7. In these specimens, the size of each transposition was quite variable with none extending to the caudal border of C7 (Figure 1b,d). It was evident in 30/44 C7s, representing 39/58 transpositions. The configuration was either a CrVT or CVT with bilateral (*n* = 7), left unilateral (*n* = 17), or right unilateral (*n* = 8).

#### 3.2.3. Descriptive Morphology of a Replicated Transverse Foramen

**Figure 2:**
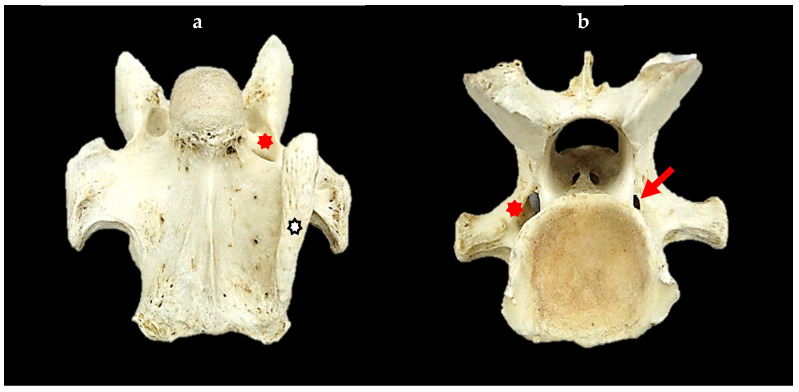
Ventral and caudal views of the seventh cervical vertebra in Tb 26. (**a**) Ventral view—left unilateral incomplete transposition (black and white asterisk) and transverse foramen (red asterisk). (**b**) Caudal view—replication of the transverse foramen, left normal in size (red asterisk) and right incomplete (red arrow), noted only in the caudal vertebral body.

The replication of a transverse foramen in C7 resembles those in C6. They vary in diameter from normal (complete) to small (incomplete). Evident in 30/44 C7s, these replications only applied to those C7s with transposition/s. The side of the replication corresponded to the side of the transposition (except in Tb 26). In these specimens, the configuration was bilateral (*n* = 13), left unilateral *(n* = 17), and right unilateral *(n* = 0), of which 40 were complete and 3 were incomplete (Figure 2).

**Figure 3:**
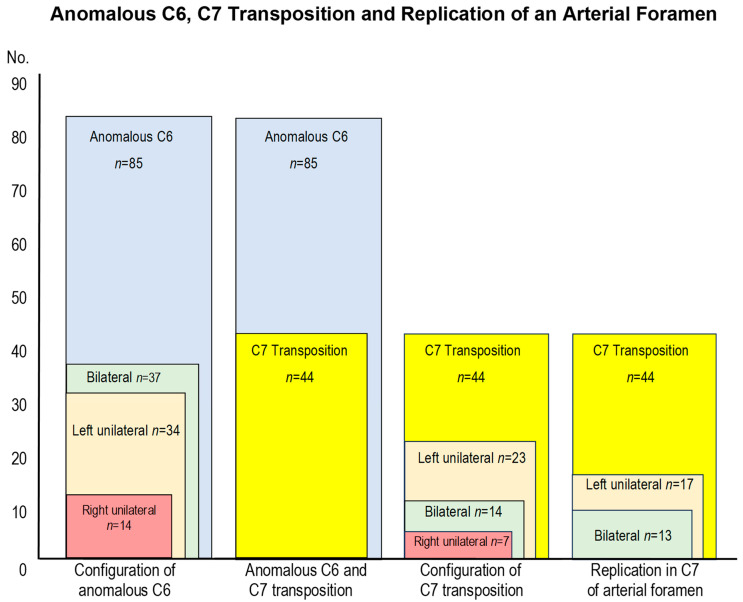
Numerical configuration of the anomalous sixth cervical vertebra, the transposition onto the ventral surface of the seventh cervical vertebra, and replication of a transverse foramen in the seventh cervical vertebra.

Overall, the most prevalent C6 and C7 morphological configuration with anomalous variations was left unilateral, then bilateral, with right unilateral the least prevalent. This configuration also applied to the replication in C7 of a transverse foramen (Figure 3).

### 3.3. Association between C6 aCVT/aCrVT Grades and C7 Transposition

As previously established [25], the aCrVT graded from 1/4 to 3/4 only, with no 4/4 grade present, and the aCrVT was only evident when the aCVT grade equated to 4/4. The associative grades between C6 aCVTs and aCrVTs and the C7 transpositions are reported in Table 1, with no differentiation between configuration (left/right unilateral or bilateral).

As indicated in Table 1, the most prevalent C7 transpositions with morphologically complete and incomplete ventral processes appeared primarily associated with C6 grades between 4/4aCVT to 3/4aCrVT.

### 3.4. Statistical Analysis

#### 3.4.1. Pairwise Associations

In the pairwise analysis using Cramer’s V coefficient for strength (0 to 1) and significance of association (*p* value), there was a stronger association between C7 transpositions and C6 aCrVT (48/58) than between C7 transpositions and C6 aCVT (10/58) when comparing the same side. Similarly, there is a strong association between C7 transposition and the transverse foramen. There is a statistically significant relationship between the variables on the same side with the exception of C6 aCVT and C7 transverse foramen, which was not statistically significant after correcting for multiple testing.

**Figure 4:**
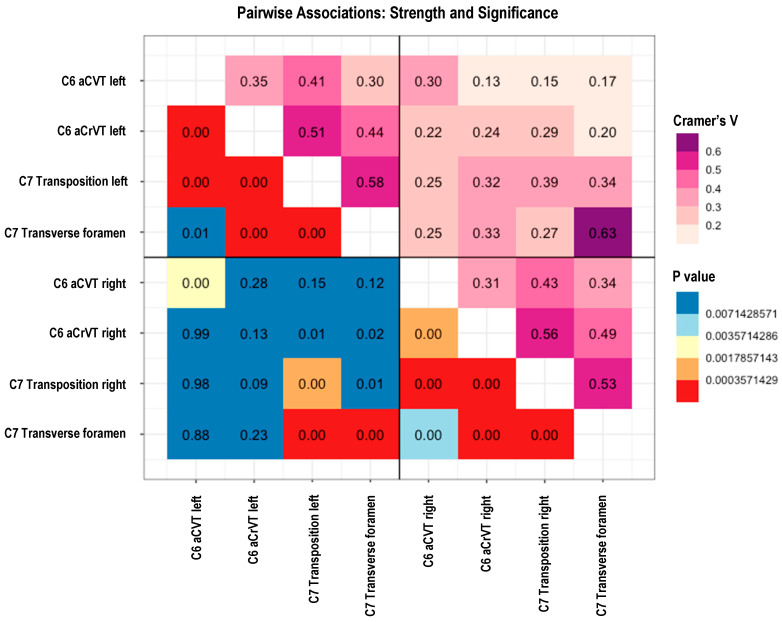
Results of pairwise associations. The values for Cramer’s V denoting strength of the association (pink to purple color palette) display weaker associations in light color shades, while stronger associations are darker. The yellow, orange, blue, and red color palette denotes statistical significance in *p* values (Fisher’s exact test) with Bonferroni correction applied.

In general, the results shown in Figure 4 suggest there is a strong dependence between abnormalities in C6 and C7 on the same side. That is, a left-sided anomalous C6 is more predictive of a left-sided transposition on the ventral surface of C7 and replication of a transverse foramen. Furthermore, anomalous variations in the left appear independent from those on the right and vice versa.

#### 3.4.2. Multiple Correspondence Analysis

With such a large dataset, and so many variables, the results of the MCA were separated into variable plots and individual plots rather than the customary biplot.

The variable plot was further separated into multiple panels by variable type and side to prevent overplotting. The MCA variable plot shows the association between the grades of absence at each location.

In the first dimension (*x*-axis), grade 4 C6 aCVTs are above 0, while normal and grades 1–3 have a value below 0. For C6 aCrVT, C7 transpositions and transverse foramen values of ‘normal’ are below 0 on the *x*-axis, while all grades of the malformation are above 0. This result shows the previously published relationship between a grade 4 C6 aCVT and a non-normal (grade 1–4) C6 CrVT. In addition, it shows the dependence between a grade 4 C6 aCVT is associated with the C7 transposition (complete or incomplete) and replicated transverse foramen.

The second dimension captures sidedness when comparing graded malformations. The left ‘normal’ generally appears higher in the *y*-axis, compared to the higher grades/abnormal values, while the right ‘normal’ appears lower in the *y*-axis.

The MCA results suggest there is a strong dependence between the anomalous C6 aCVT and the C6 aCrVT, and additionally, this dependence extends from the anomalous C6 malformations to the transposition onto the ventral surface of C7 of either a complete or incomplete ventral tubercle/process and replication of the transverse foramen.

### 3.5. Incidental Findings

During the dissection of several animals and the examination of the associative C7 morphology, the following incidental findings were noted.

**Figure 5:**
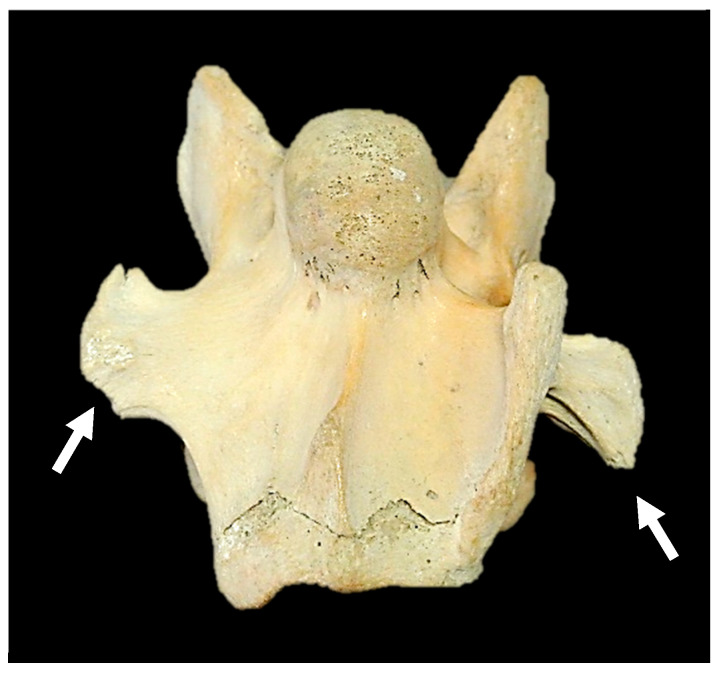
Ventral view of the seventh cervical vertebra with asymmetric transverse processes (white arrows) in Tb 2.

1. No tendon was evident at the attachment of the longus colli (thoracal portion) to C6 in grade 4 aCVT and grades 1–3 aCrVT.
2. Hypertrophy was noted in the belly of the above longus colli muscle.
3. The scalene muscles (dorsal and ventral) could bifurcate at the first sternal rib attachment with cervical nerves from the brachial plexus traversing through either bifurcation as opposed to the normal path between the dorsal and ventral scalene muscles.
4. The vertebral artery passes through C1–C6. Yet, in the replication of the transverse foramen in C7, this artery leaves the subclavian artery and passes the cranial to the facies articularis capitis costae (cranial) of the first sternal rib caudal to C7 and enters the replicated transverse foramen in C7 caudally before traversing towards the transverse foramen of C6 thereafter.
5. The ventral surface of C7 was representational of C6 in a bilateral complete presentation, along with bilateral transverse foramen, a flattened sagittal ridge, and no ventral crest.
6. Several C7 osseous specimens were deficient in fovea costalis caudalis corresponding to the side of the transposition whether complete or incomplete.
7. The transverse processes of C7 in a unilateral transposition were often asymmetrical, and this morphology also applied in asymmetrical bilateral transpositions (Figure 5).

## 4. Discussion

The mounting concern in the horse community of congenital malformations in the cervicothoracic region known as ECVM has a plethora of equine researchers examining the complexities associated with this condition [11,12,13,14,15,16,17,18,20,21,22,23,25,29,34,35]. This manuscript examined one such complexity not yet studied, notably, the association in grades of C6 aCVTs and aCrVTs to the anomalous transposition, whether complete or incomplete, on the ventral surface of C7 and the replication of transverse foramen.

Originally, when examining the C7 transposition on osseous specimens, it was described by some as an additional prolongation [10], bony prominence [35], transposed CVT [12], or by radiographs as a bony protuberance [29]. These reports all have merit, yet when assessing radiographs, it would appear the interpretation is logical if only the cranial portion of C7 is visible due to the caudal view being partially obscured by the scapula in some horses. Even so, May-Davis (2014) described the morphology from osseous specimens [12] and referred to the transposition as a CVT commensurate with C6. In contrast, the statistical analysis in the current study suggests the more prevalent anomalous transpositions were representational of an aCrVT (39/58) and not CVT, as reported by May-Davis (2014) [12]. That is to say, the complete transpositions were morphologically mimicking the ventral process from C6 on the ventral surface of C7. Yet, there was no statistical association as to which C6 grade aCVT and or aCrVT reflected upon the type of transposition (complete or incomplete) identified on C7. Although sidedness was recognized between C6 and C7, the left-sided anomalous C6 was more predictive of a transposition on the left ventral surface of C7 than in bilateral or right-sided cases.

When Ros et al. (2023) [36] graded the C7 transposition from 1–3 via radiographs, it was an ascending scale according to size. In the current study, the grades 1–2 by Ros et al. (2023) correlate with an incomplete transposition, while a grade 3 represents a complete transposition. To date, the two primary protocols for radiographing the C7 transposition [21,36] show a CrVT-like structure projecting cranially on the ventral surface of C7, which is virtually identical to the morphology of a C6 CrVT in radiographs. Hence, as identified by Gee (2020) [21], C7 might be confused for a C6 when a C6 grade 4 aCVT and or 1–3 grade aCrVTs are present and C6 is representational of C5, especially in bilateral specimens. The newer protocols for radiographing C7 by Ros et al. (2023) [36] might help alleviate the concerns established by Gee (2020) [21]. Yet, it is worthwhile to note there is a statistically stronger dependence between C6 grades 1–3 aCrVT and C7 transposition with transverse foramen than C6 aCVT grades. Furthermore, there is a strong dependence between an anomalous C6 and corresponding anomalous C7 being ipsilateral. Therefore, further investigation of C7 might be warranted if a C6 grade 4 aCVT and/or C6 grade 1–3 aCrVTs are radiographically evident.

As reported in the incidental findings, these transpositions coincide with *longus colli* variations, confirming May-Davis and Walker’s (2015) previous findings [22]. Yet, the bifurcation of the scalene muscles, relocation of brachial plexus nerves, and subsequent pathways might explain some of the clinical concerns raised by previous authors when the patient appears symptomatic of neck pain, thoracic limb ataxia, and/or forelimb paresis and paresthesia [16,17,22,23,29]. These concerns might also include the absent *fovea costalis caudalis*, relative to the first sternal rib, where associative malformations have previously been reported [13,23,36], and the asymmetric transverse processes through uneven biomechanical load. Furthermore, the question must be asked if the larger complete transpositions with transverse foramen interfere with other structures at the point of entry into the thoracic inlet where the vertebral artery might also be compromised. These complex associative variations are yet to be thoroughly explored, especially the relevance to clinical findings and surgical procedures in the region, which to date have not yet been fully investigated [29].

In 2015, May-Davis and Walker described a bilateral C6 female and her bilateral C6 spontaneously aborted 9-month male foal and suggested a maternal link [22]. Zimmerman et al. (2023) hinted that male ancestral sires with anomalous C6 and C7 with unilateral and bilateral malformations might provide a mode of inheritance in Thoroughbred and modern German Warmblood horses [34]. Similar studies of wolves (*canis lupis*) in two separate populations reported anomalous C6 and C7 congenital malformations like that described here [37]. Both studies detected inbreeding and genetic deterioration in the population. If a mode of heritability does exist in the equine studies, does this equate to the other anomalous variations noted in the incidental findings? Unfortunately, the scope of this study was too limited to address this question and those pertaining to the bloodlines of each individual, but it is worthwhile noting that the largest demographic in this study belonged to Thoroughbreds and Warmbloods from multiple geographical locations, suggesting a familial and or genetic link as per that proposed by Zimmerman et al. (2023) [34]. The likelihood of such a global influence might be seen in the use of shuttle stallions in the Thoroughbred industry [38] and artificial insemination (AI) in Warmbloods [39]. AI is the known propagator of CVM in Holstein cattle [40], suggesting this mode of delivery might also be responsible for the C6/C7 anomalous trait in many breeds of modern horse today.

Of particular importance are the results from experimental studies on mice that show cervical malformations from C6 to T1 develop during embryogenesis as a result of targeted disruption to specific Homeobox (Hox) genes. These studies concluded the experimentally induced malformations were consistent with anterior homeotic transformations [41,42,43,44,45,46,47]. Following Bateson (1894), homeotic transformation occurs when “*one of the component parts of the axial skeleton assumes the morphological appearance and function of its neighbor either immediately preceding or immediately following it.*” [48]. The morphology of these malformations in mice C6 and C7 vertebrae are nearly identical to those characteristics expressed in the osseous presentations reported in ECVM cases [42,43,44,45]. Moreover, as the grading system of C6 indicates, left and right sides within the same cervical vertebra can have different grades [25]. This correlates with Rancourt (1995), who described similar cervical malformations resulting from targeted mutations in mice that were restricted to, or were more severe on, one side than the other of C6–C7 [43]. He proposed a “progression in stages of transformation” due to variability in expressivity of the targeted Hox genes with the end stage being complete transformation.

Ultimately, this study indicates that more research is necessary to understand the morphological nuances associated with both the phenotypic and genotypic etiology driving these malformations.

## 5. Conclusions

This study demonstrated an association between the size of the absent ventral process on C6 and transposition/s on the ventral surface of C7 with transverse foramen. A strong dependency was evident between the C6 aCVT grade 4 through to aCrVT grades 1–3 and the C7 transposition/s with replicated transverse foramen. These findings supported previous studies whereby anomalous transpositions onto the ventral surface of C7 concurred with absent ventral tubercles(s) of C6. Furthermore, sidedness was evident in transpositions, while breed dependency or geographical location was not. Of concern is the change in morphological characteristics of C6 to that of C5 and from C7 to that of C6, often referred to as homeotic transformation. This would require further investigation into the genetic components influencing these congenital malformations, especially when evidenced that deliberate manipulation of specific Hox genes can create similar malformations in the caudal cervical vertebrae of mice.

## Figures and Tables

**Table 1:** Associative C7 transpositions (complete and incomplete) transposed ventral processes relative to the grade of the C6 absent caudal ventral tubercle (aCVT) and absent cranial ventral tubercle (aCrVT).

No Transposition	7	11	8	39	5	7	0
Complete	0	0	0	2	3	10	4
Incomplete	0	0	2	6	12	15	4
Total transpositions	0	0	2	8	15	25	8
Grade C6	1/4 aCVT	2/4 aCVT	3/4 aCVT	4/4 aCVT	1/4 aCrVT	2/4 aCrVT	3/4 aCrVT

## Data Availability

Data are contained within the article and supplementary materials.

## Supplementary Materials

The following supporting information can be downloaded at: https://www.mdpi.com/article/10.3390/ani14121830/s1, Supplementary Table S1: Individual details of the 85 osseous specimens that displayed an absent caudal ventral tubercle of the sixth cervical vertebra with grading, complete (c) and incomplete (In) transposition onto the ventral surface of C7 and replication of the arterial foramen.
