# Supplementary material for: Characterization and Association of the Missing Ventral Tubercle(s) from the Sixth Cervical Vertebra and Transpositions on the Ventral Surface of the Seventh Cervical Vertebra in Modern Equus ferus caballus
Source: Animals (Basel). 2024 Jun 20;14(12):1830. doi: 10.3390/ani14121830 (PMC11200614; doi:10.3390/ani14121830)
Supplement: Supplementary file 1 [file animals-14-01830-s001.zip › animals-3026374-supplementary.pdf]

Supplementary Table S1. Individual details of the 85 osseous specimens that displayed an absent caudal ventral tubercle of the sixth cervical vertebra with grading, complete (c) and incomplete (In) transposition onto the ventral surface of C7, and replication of the arterial foramen.

Breed ID	County	Age	Discipline	Years ridden	Height range	Sex	C6 aCVT Grade 1-4		C6 aCrVT Grade 1-3		C7 Transposition		Arterial Foramen	
							Left	Right	Left	Right	Left	Right	Left	Right
Tb 1	Aus	5	racing		>15	M	4		2		In		C	
Tb 2	Aus	6	racing	3	>15	M	4		2		In		C	
Tb 3	Aus	4	racing	2	>15	M	4		3		In		C	
Tb 4	Aus	12	R/R	15	>15	F	3	4						
Tb 5	Aus	22	R/R	10	>15	F	4	4						
Tb 6	Aus	18	End	6	>15	M		2						
Tb 7	Aus	8	Br		>15	F	4	4	2	1	In	In	In	In
Tb 8	Aus	6	racing	3	>15	M	4		2					
Tb 9	Aus	5	racing	2	>15	F	4		2		In		C	
Tb 10	Aus	12	Event	8	>15	F		4						
Tb 11	Aus	6	racing	3	>15	F	4		2		In		C	
Tb 12	Aus	10	R/R	7	>15	M	4	4	1	1	In	In	C	C
Tb 13	Aus	16	Event	13	>15	F	4	4	3	3	In	In	C	C
Tb 14	Aus	23			>15	M	4		2					
Tb 15	Aus	10mths				M		4		2		In		
Tb 16	Aus	8	R/R	5	>15	F	4	4			In	In	C	C
Tb 17	Aus	12	R/R	9	>15	M	2							
Tb 18	Aus	18			>15	M	4	4						
Tb 19	Aus	3			>15	M	4	4						
Tb 20	Aus	12			>15	M		1						
Tb 21	Aus	22	R/R	19	>15	F	4	4						

Tb 22	Japan	23	R/R	20	>15	M	3				In		C	
Tb 23	Japan	22	R/R	19	>15	M	4		1					
Tb 24	Japan	17	R/R	14	>15	F		4		2				
Tb 25	Japan				>15	M	4		1		In		C	
Tb 26	Japan	7			>15	F	4	4			In		C	In
Tb 27	UK	16	R/R	13	>15	M	4		2		In		C	
Tb 28	UK	12			>15	M	3							
Tb 29	Ireland	13	racing	9	>15	M	4	3						
Tb 30	NZ	8	racing		>15	F	4	3						
Tb 31	USA	22	R	19	>15	F	3							
Tb 32	Aus	6			>15	M	4	4	2	2	C		C	
Tb 33	USA	9			>15	F	4		2		In		C	
Tb 34	USA	9	R/R		>15	M	4		1		In			
Tb 35	USA	16			>15	M	4	4	1	1	In	In	C	C
Tb 36	USA	12			>15	M	1							
Tb 37	USA	24			>15	F	4							
Tb 38	USA	5	R/R		>15	M	4	4	2	2	C	C	C	C
Tb 39	USA	20+			>15	F		4				In		
Tb 40	USA	13	R/R	10	>15	M	4		3		C		C	
Tb 41	Aus	12	R/R	9	>15	M		4				In	C	C
Tb 42	UK	19	Eventing	16	>15	M	4	4	2			In		
Wb 1	Belgium	SB				M	4	4	2	2	C	C	C	C
Wb 2	Aus	15	R	5	>15	F	4		2		In		C	
Wb 3	Netherlands	18	R	6	>15	F		2						
Wb 4	Netherlands	13	R	10	>15	F	2							
Wb 5	Aus	6	R	3	>15	F	1							
Wb 6	Netherlands	2	R		>15	F	4		2		In		C	

Wb 7	Aus	23	R		>15	M	4	4	3	2	C	In		
Wb 8	Aus	9	R		>15	F		4						
Wb 9	Aus	14	R	11	>15	M	3							
Wb 10	Aus	6	R		>15	M	4	4	1					
Wb 11	USA	8			>15	M	4		1		In		C	
Wb 12	USA	17			>15	M	4	4		1		In		
Wb 13	USA	6	R	3	>15	F	4	3	1					
Wb 14	USA	10	R	6	>15	M		4		1				
Wb 15	USA	15	R	12	>15	F	4	4	2	2	In	In	C	C
ASH 1	Aus	SB				F	4	4						
ASH 2	Aus	3	R	3 months	14-15	M	4	4	2	2				
ASH 3	Aus	22	R		>15	F	4		2		In		C	
ASH 4	Aus	10	R		>15	M	4		2					
ASH 5	Aus	8	R		>15	F	4		2		C			
ASH 6	Aus	5	R	2	14-15	M		4				C		
XB 1	Aus	30	R	20	14-15	M	4		3		In		C	
XB 2	Aus	12	R	9	14-15	F	4	1	2					
XB 3	Aus	SB				M	4	4						
XB 4	Aus	15	R	10	14-15	M		3						
XB 5	Aus	8	R	5	14-15	M	2	4		1		In		
XB 6	Aus	5	R	2	>15	M	4		1		In			
Sb 1	Sweden	7	H	5	>15	M		2						
Sb 2	Aus	13	H/R	8	>15	M	1							
Sb 3	NZ	22	H		>15	M	2							
Sb 4	Aus	12	H		>15	M	2							
Sb 5	Aus	4	H	1	>15	M	4		1					
App 1	USA	23				F	4	4	2	1	C	C	C	C

App 2	USA	22				F	4	1	1		C			
App 3	USA	35				M	4	4						
RP 1	Aus	8	R		<14	M	4	3	1		C	In		
RP 2	Aus	16	R	13	<14	M	4	4	2	3	C	C	C	C
RP 3	Aus	7	R	4	<14	M	1	2						
QH 1	Aus	7	R			M	4		2		C		C	
QH 2	USA	28				M	4	2			C			
QH 3	USA	12				F	4	4	1	2	In	In		
Friesian	USA	10			>15	M	4	4	2	3	C	C	C	C
ISH	UK	5	R	2	>15	M		2						

**Key**
In - Incomplete
C - Complete
R/R - racing and riding
End - endurance
Br - broodmare
Event - eventing
H - harness
H/R - harness & riding
F - female
M - male
SB - still born

**Key**
Tb - Thoroughbred
Wb - Warmblood
ASH - Australian Stock Horse
XB - Crossbred
Sb - Standardbred
App - Appaloosa
RP - Riding Pony
QH - Quarter Horse
ISH - Irish Sport Horse
Aus - Australia
UK - United Kingdom
NZ - New Zealand
USA - United States of America
